# Managerial leadership for research use in nursing and allied health care professions: a systematic review

**DOI:** 10.1186/s13012-018-0817-7

**Published:** 2018-09-27

**Authors:** Wendy A. Gifford, Janet E. Squires, Douglas E. Angus, Lisa A. Ashley, Lucie Brosseau, Janet M. Craik, Marie-Cécile Domecq, Mary Egan, Paul Holyoke, Linda Juergensen, Lars Wallin, Liquaa Wazni, Ian D. Graham

**Affiliations:** 1 Faculty of Health Sciences, School of Nursing, University of Ottawa, Ottawa, Ontario Canada; 2 Clinical Epidemiology Program, The Ottawa Hospital Research Institute, Ottawa, Ontario Canada; 3 Telfer School of Management, University of Ottawa, Ottawa, Ontario Canada; 4 Canadian Nurses Association, Ottawa, Ontario Canada; 5 Faculty of Health Sciences, School of Rehabilitation Sciences, University of Ottawa, Ottawa, Ontario Canada; 6 Canadian Association of Occupational Therapists, Ottawa, Ontario Canada; 7 Health Sciences Library, University of Ottawa, Ottawa, Ontario Canada; 8 Elisabeth Bruyere Research Institute, Ottawa, Ontario Canada; 9 SE Research Centre, SE Health, Markham, Ontario Canada; 10 Faculty of Health, School of Nursing, York University, Toronto, Ontario Canada; 11 School of Education, Health and Social Studies, Dalarna University, Falun, Sweden; 12 Sahlgrenska Academy, Department of Health Care Sciences, University of Gothenburg, Gothenburg, Sweden; 13 Department of Neurobiology, Care Sciences and Society, Division of Nursing Karolinska Institutet, Stockholm, Sweden; 14 School of Epidemiology and Public Health, University of Ottawa, Ottawa, Ontario Canada

**Keywords:** Leadership, Managers, Administrators, Research use, Evidence-based practice, Allied health, Nursing

## Abstract

**Background:**

Leadership by point-of-care and senior managers is increasingly recognized as critical to the acceptance and use of research evidence in practice. The purpose of this systematic review was to identify the leadership behaviours of managers that are associated with research use by clinical staff in nursing and allied health professionals.

**Methods:**

A mixed methods systematic review was performed. Eight electronic bibliographic databases were searched. Studies examining the association between leadership behaviours and nurses and allied health professionals’ use of research were eligible for inclusion. Studies were excluded if leadership could not be clearly attributed to someone in a management position. Two reviewers independently screened abstracts, reviewed full-text articles, extracted data and performed quality assessments. Narrative synthesis was conducted.

**Results:**

The search yielded 7019 unique titles and abstracts after duplicates were removed. Three hundred five full-text articles were reviewed, and 31 studies reported in 34 articles were included. Methods used were qualitative (*n* = 19), cross-sectional survey (*n* = 9), and mixed methods (*n* = 3). All studies included nurses, and six also included allied health professionals. Twelve leadership behaviours were extracted from the data for point-of-care managers and ten for senior managers. Findings indicated that managers performed a diverse range of leadership behaviours that encompassed change-oriented, relation-oriented and task-oriented behaviours. The most commonly described behavior was support for the change, which involved demonstrating conceptual and operational commitment to research-based practices.

**Conclusions:**

This systematic review adds to the growing body of evidence that indicates that manager-staff dyads are influential in translating research evidence into action. Findings also reveal that leadership for research use involves change and task-oriented behaviours that influence the environmental milieu and the organisational infrastructure that supports clinical care. While findings explain how managers enact leadership for research use, we now require robust methodological studies to determine which behaviours are effective in enabling research use with nurses and allied health professionals for high-quality evidence-based care.

**Trial registration:**

PROSPERO CRD42014007660

**Electronic supplementary material:**

The online version of this article (10.1186/s13012-018-0817-7) contains supplementary material, which is available to authorized users.

## Background

The use of research evidence in clinical practice has advanced healthcare delivery from unpredictable and unproven practices to treatments based on rigorous research evidence to improve outcomes [1, 2]. However, research use continues to be a challenge across all healthcare disciplines and settings [3–5], with over two-thirds of implementation efforts deemed unsuccessful [6]. For example, a recent cross-sectional survey revealed that only 12% of nurses and allied health professionals in the European Society of Cardiology used research-based evidence from guidelines in their practice [7]. While much of the implementation research focusses on individual practitioners [8], leadership within the organisational context is increasingly recognized as a strong influencing factor on the acceptance and use of research evidence in practice [9]. In the present study, the concept of using research in clinical practice is based on Sackett et al.’s (1997) widely accepted definition of evidence-based medicine: ‘the conscientious, explicit and judicious use of current best evidence in making decisions about the care of individual clients’ [10].

With the growing recognition of the importance of leadership in implementation efforts, the mechanisms by which leadership influences research use are receiving increasing attention [9, 11]. Leadership has been defined and studied in many ways across disciplines. In this study, we use a highly used definition of leadership as a process that influences, motivates, and enables others [12]. Behavioural leadership theory suggests that effective leadership involves behaviours from three broad conceptual categories: (1) change-oriented, (2) relation-oriented and (3) task-oriented behaviours [13–15]. Change-oriented behaviours are concerned primarily with providing vision and direction for innovation, creating a sense of need, and building coalitions to support change. Relation-oriented behaviours involve supporting, developing and recognizing others with the primary objective to increase the quality of human resources and relations, thereby increasing trust, cooperation and commitment amongst members. Task-oriented behaviours include clarifying roles, planning, monitoring performance and outcomes and using resources efficiently [13–15].

Transformational and transactional leadership theories are well known and widely researched leadership approaches [16, 17]. Transformational leadership is the degree to which a leader inspires and motivates others to follow an ideal or a particular course of action [16], while transactional leadership involves the provision of incentives, rewards and monitoring to meet quality standards [17]. Dimensions of both transformational and transactional leadership align with the leadership behaviours in task-oriented, relation-oriented and change-oriented conceptual categories. For example, transformational leadership can influence attitudes towards research use through relations and change-oriented behaviours of envisioning change, facilitating collective learning and supporting and recognizing efforts, whereas transactional leadership aligns with task-oriented behaviours of clarifying roles, planning and monitoring operations to accomplish work in an efficient and reliable way. Consistent with behavioural leadership theory, the relevance of each behavior depends on the aspect of the situation and the context of the implementation efforts [13].

The leadership behaviours of point-of-care and senior managers have been shown to strongly influence nurses and allied healthcare professionals’ use of research evidence, while lack of leadership is consistently identified as a major barrier to implementation [18–21]. Managers are employees who oversee staff, have budgetary accountabilities [22] and play a role in ensuring high-quality patient care [23, 24]. Point-of-care managers (e.g. head nurses, managers or supervisors) are responsible for unit operations, with front-line staff reporting to them, while senior managers (e.g. administrators, directors, operating officers) have broader organisational responsibilities, with one or more managers typically reporting to them [25]. A recent American mixed-methods study examining implementation of an evidence-based innovation in social welfare organisations to reduce child maltreatment found that successful implementation was 17 times higher with strong leadership, and failure was associated with passive/avoidant leadership [9].

Nurses and allied healthcare professionals constitute the largest proportion of the healthcare team and play a central role in ensuring high-quality and effective care delivery. Nurses are self-regulated professionals that deliver autonomous and collaborative care in health promotion, illness prevention and caring for ill, disabled and dying people [26]; they include registered nurses (RNs), licensed practical nurses (LPNs), registered practical nurses (RPNs), nurse practitioners and registered psychiatric nurses [27]. Allied health professionals are licenced to provide specific types of healthcare services but are not physicians or nurses [28]. While disciplines under the umbrella term ‘allied health’ vary [29], for purposes of this review they include physiotherapists (PTs), occupational therapists (OTs), speech-language pathologists (SLPs) and dietitians as defined a priori in the study protocol [30].

Managers are strategically positioned to support and facilitate nurses and allied health professionals’ use of research evidence through organisational policies, procedures, systems and climates [9, 31]. A 2007 integrative literature review identified that managers used facilitative and regulatory behaviours to influence nurses to use research evidence, including support, policy revisions and clinical practice audits [32]. However, relevant literature has not been systematically synthesized for allied health professionals or updated for nurses, and little is known about healthcare managers’ approaches to support their research use. Understanding leadership behaviours that advance research use is fundamental for designing interventions for organisations to improve healthcare delivery and patient outcomes.

The purpose of this systematic review was to synthesize evidence on the association between leadership behaviours of point-of-care and senior managers and research use by nurses and allied health professionals. The specific objectives were (1) to identify managers’ leadership behaviours that are associated with research use by nurses and allied health professionals in clinical practice and, if studies permit, (2) to determine the effectiveness of interventions to develop leadership for facilitating research use by nurses and allied health professionals.

## Methods

We conducted a mixed-methods systematic review to synthesize diverse forms of evidence related to point-of-care and senior managers’ leadership behaviors that are associated with nurses and allied health professionals’ research use in clinical practice [30]. We used a systematic approach to synthesize quantitative, qualitative and mixed-methods results using methodological guidelines set forth by Grimshaw [33].

### Concepts and definitions

Several forms of research use have been discussed in the literature, including instrumental, conceptual and symbolic [34, 35]. We focussed on instrumental research use or the concrete application of research knowledge as we were interested in improved healthcare delivery through behaviour change in clinical practice. The *evidence* included guidelines, protocols, policies or procedures based explicitly on research. We defined leadership ‘behaviours’ as managerial activities and engagement practices that influence nurses and/or allied health providers to use research evidence in their clinical practice.

### Search strategy

In collaboration with a health sciences librarian, we developed and implemented the search strategy, using eight electronic bibliographic databases (ABI Inform Global, CINAHL, Cochrane Database of Systematic Reviews, Cochrane Central Register of Controlled Trials, EMBASE, MEDLINE, Pedro, Proquest Nursing and Allied Health, PsycINFO) and covering all available published works up to June 2018. Keywords, and their synonyms and medical subject headings were used for leadership, management and research use in each database (see Additional file 1 for search strategy). Reference lists of included studies were assessed for relevant citations.

### Study inclusion/exclusion criteria

Studies investigating managerial leadership behaviours and staff research use were included. To be included, studies needed to report on actual (not planned) instrumental research use and managerial leadership behaviours. Instrumental research use was expressed at the individual practitioner or unit levels and included settings classified as having high and low levels of research use. Evidence-based practice [36] was included if instrumental research use was studied separately from the multi-step process of constructing a clinical question and critically appraising the literature. Studies were excluded if leadership could not be clearly attributed to someone in a management position such as those using the terms ‘leader’, ‘senior nurse’, ‘hospital leadership’ or ‘organisational leadership’ without identifying a management role, or if greater than 50% of the sample was not nurses or allied health professionals. Studies were limited to those published in English or French, the official languages of our research team, with no restrictions on country of origin or publication date.

#### Types of studies

Experimental (e.g. randomized controlled trials), quasi-experimental (e.g. pre/post-test), non-experimental (e.g. cross-sectional surveys), mixed-methods and qualitative designs were included. Commentaries, editorials and theses were excluded.

Quantitative studies had to propose a relationship between managerial leadership behaviours and staff research use and test it statistically, with instrumental research use as the dependent variable and leadership as the independent variable. Interventions must have involved front line or senior level managers for the purpose of influencing clinical staff use research in practice.

#### Participants

Nurses included RNs, LPNs, RPNs, nurse practitioners and registered psychiatric nurses; allied health professionals included PTs, OTs, SLPs and dieticians.

### Selection of studies

Two reviewers independently screened all titles and abstracts identified in the database search for eligibility. Full-text copies were retrieved for all citations identified as potentially relevant or having insufficient information to make a decision. Retrieved articles were assessed for alignment with inclusion criteria independently by two team members; discrepancies were resolved through discussion and adjudication with senior research team members (WG, JES, IDG).

### Quality assessment

We used three tools to assess the methodological quality of included studies according to study design: (1) *Quality Assessment and Validity Tool for Cross-sectional Studies*, (2) *Quality Assessment and Validity Tool for before/after Design studies* and (3) *Critical Appraisal Skills Programme (CASP) Qualitative Research Checklist.* Two reviewers independently conducted the quality assessment on all included articles; disagreements were resolved through discussion with a third senior reviewer. We adopted a scoring system used in a previously published systematic review [37]: for each article, a rating score was derived by taking the number obtained in the quality rating and dividing it by the total number of possible points allowed, giving each paper a total quality rating between 0 and 1. Articles were then classified as weak (< 0.50), moderate-weak (0.51–0.65), moderate-strong (0.66–0.79) or strong (0.80–1.00). Mixed-methods studies were assessed with two corresponding tools.

Qualitative studies were assessed using the Critical Appraisal Skills Programme (CASP) Qualitative Research Checklist [38], which assesses methodology through ten questions on research aims, appropriateness of methodology, research design, recruitment strategy, data collection, adequate consideration of the relationship between researchers and participants, ethical issues, data analysis, clarity of findings and research value. Cross-sectional quantitative studies were assessed with the Quality Assessment and Validity Tool for Cross-sectional Studies [39], which focuses on reporting quality and methodological rigor in four domains: sample, measurement, statistical analysis and conclusion. Intervention studies were assessed using the Quality Assessment and Validity Tool for before/after Design studies, adapted from Cochrane Collaboration guidelines and used in other systematic reviews [40]. It focuses on six domains: sampling, design, control of confounders, data collection and outcome measurement, statistical analysis and conclusions as well as dropouts. No studies were excluded based on the quality assessment.

### Data extraction

One reviewer extracted data from all included articles, a second reviewer verified for accuracy and a senior reviewer resolved discrepancies. Data were extracted on publication year, country, research purpose and objectives, research design, setting, data collection methods, sample size and participant characteristics, behaviours of managers (independent variables/concepts), managers’ titles and characteristics, research use variables or concepts, analysis, and key findings regarding the relationship between managers’ leadership behaviours and research use by nurses and allied health professionals.

### Data synthesis

A narrative data synthesis was conducted using Popay et al.’s [41] procedures to produce a summary of the research studies. Qualitative study data on managers’ behaviours were pooled and entered into NVivo qualitative software then inductively coded into descriptive themes using the primary authors’ conceptualizations of the behaviours described. For example, if an author reported ‘encourage’ as a managerial behaviour, it was classified as ‘encourage’ in our analysis and not reclassified based on interpretations. We used a consensus technique to determine the descriptive themes and made inquiries to primary authors of included studies to clarify interpretations when needed.

Data extracted from quantitative studies were synthesized descriptively, identifying the dependent (research use) and the independent (leadership) variables. This was supplemented by extracting the direction and magnitude of effect for factors displaying statistical significance (*p* < 0.05) where provided. Additionally, when bivariate and multivariate statistics were both reported, the more robust multivariate findings were used.

Quantitative data were synthesized into descriptive themes using convergence when data from the two methods corroborated and expansion when additional insights were provided. The use of different leadership measures in the small number of quantitative studies prevented quantitative data from being combined for sub-group analysis or statistical assessment of the association between managers’ leadership and research use.

Based on behavioral leadership theory, descriptive themes were deductively grouped into the three conceptual domains of leadership: change-oriented, relation-oriented and task-oriented leadership behaviours [12–14]. Data categorization was initially completed by the research assistants and first author (WG); further synthesis and re-classifications occurred iteratively in group meetings with investigators (WG, IDG, JES, LW), where study data were compared and contrasted with descriptions of the behavioral leadership categories [14]. Findings were discussed with the entire research team until consensus was reached.

We did not analyze studies for the effectiveness of leadership interventions on research use by nurses and allied health professionals (objective two) because of the lack of experimental studies found in the review. The limited number of studies found also prevented us from conducting sub-group analysis for professional group, sector, or types of instrumental research use (i.e. research use or guideline use). While insufficient evidence was found to reach definitive conclusions regarding leadership behaviours associated with research use, findings from all included studies were narratively synthesized to provide a summary of the types of behaviours studied.

## Results

**Fig. 1:**
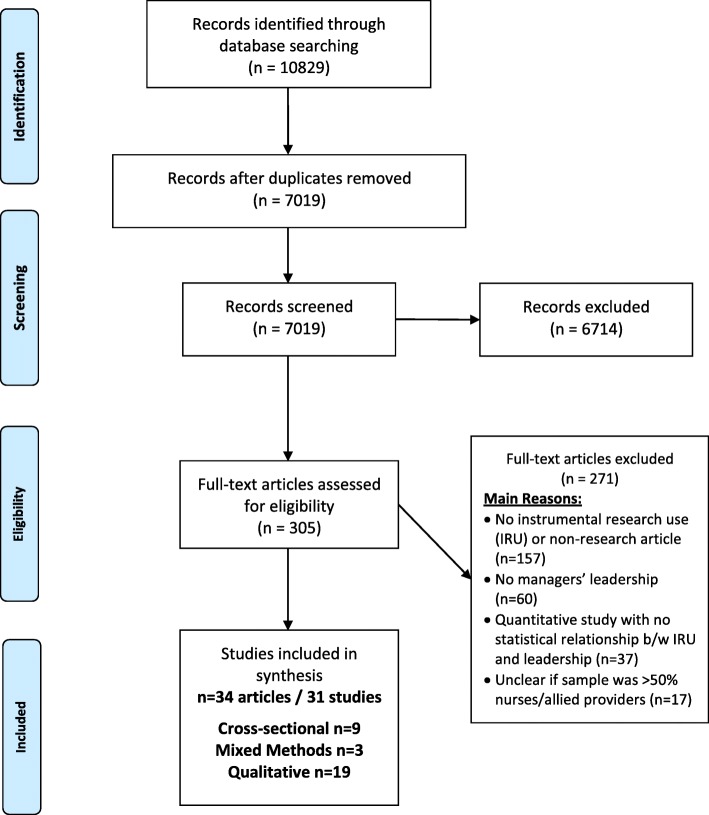
PRISMA diagram of study selection

The database search yielded 7019 unique titles and/or abstracts after duplicate removal, with 305 identified as potentially relevant and retrieved in full text. Of those, 271 did not meet our inclusion criteria: 158 lacked instrumental use of research evidence in clinical practice by nurses or allied health professionals (for example studies about barriers and facilitator pre-implementation), 60 did not have behaviours performed by managers, 37 quantitative studies had no statistical evaluation of instrumental research use and a leadership measure and 17 had a sample with < 50% nurses or allied health professionals. Thirty-one studies represented in 34 articles met our inclusion criteria and were included in this review (Fig. 1).

### Description of studies

Of the 31 studies, 9 reported cross-sectional surveys [42–50], 3 had mixed-methods designs [31, 51, 52], and 19 had qualitative designs [5, 53–73]. The mixed-methods studies included qualitative data with either a survey, experimental pilot, or quasi-experimental trial. Studies were conducted in Canada (*n* = 14) [31, 46, 48, 53–60, 66, 68, 70], Sweden (*n* = 6) [43–45, 47, 62, 73], USA (*n* = 5) [5, 42, 51, 52, 69], China (*n* = 1) [63, 64], Mongolia (*n* = 1) [65], Netherlands (*n* = 1) [71] and one study in multiple European countries [61]. Studies had different healthcare settings with 18 conducted in acute care hospitals [5, 27, 42, 46–49, 52–54, 56–59, 62–64, 67, 69, 72, 73], three in nursing homes [50–52], three in the community [31, 43, 44], one in each family health centre [65] and rehabilitation centre [70] and five had a combination [45, 55, 60, 68, 71]. All studies included nurses as participants with 20 exclusively targeting nurses (65%), six also included allied health practitioners [43, 51, 57, 60, 67] and eight also included other health providers such as physicians [51, 58, 59, 63–65, 68, 71, 72] and healthcare aids [50, 68]. Characteristics of included studies are shown in Table 1.

**Table 1:** Characteristics of included studies

First author, year, journal	Design	Sample/subjects*	Setting/country	Type of research use (DV measured by)	Manager level	Leadership measured by	Bivariate • Test statistic Magnitude (if significant)	Multivariate • Test statistic Magnitude (if significant)	Leadership behaviors studied	Quality assessment (weaknesses)
Quantitative studies (*n* = 9)										
1. Ball 2010 [42] Association of perioperative Nursing (AORN)	Cross-sectional survey	*N* = 777 Nurses	Hospital USA	Level of compliance with research-based guideline recommendations for smoke evacuation practices for 8 surgical procedures in an operating room	Senior	Three items from a survey developed for the study: Support for clinical practices^1^ Support for smoke evacuation practices^1^ Support for policies and procedures^1^	ANOVA *F* (2609) = 4.423 *p* = .012 + (magnitude not reported) *F* (2585) = 11.043 *p* < .001 + (magnitude not reported) *F* (2584) = 10.481 *p* < .001 + (magnitude not reported)		• Support the change • Embed practices in policy	High moderate -Self report -Management of missing data not reported -Response rate < 50%
2. Boström 2007 [43] Journal of Evaluation in Clinical Nursing	Cross-sectional survey	*N* = 132 Nurses Allied Healthcare Aides	Community Sweden	Single item from Research Utilization Questionnaire - ‘I use research findings in my daily practice’ scored on a 5-point Likert scale^2^ Sample divided into 2 groups: research users and non-research users	Point-of-care	One item from the Research Utilization Questionnaire: Support from unit manager^2^	Chi square (χ^2^ = 22.194)* p < 0.001 + 50% more in research group (64 vs. 14%) *Proportion of nurses in RU group versus non-RU that agree there is leadership support	Logistic regression OR = 4.03; 95% CI = 1.04–15.71 (controlling for access to research findings; challenge, support from colleagues, trust, risk-taking)	• Support the change	High moderate -Self report -Dependent variable reliably and validly not reported
3. Boström 2009 [44] Journal of Clinical Nursing	Cross-sectional survey	*N* = 210 Nurses	Community Sweden	Nine item index for ‘research use in daily practice’ from the Research Utilization Questionnaire	Point-of-care	One item from the Research Utilization Questionnaire: Support from unit manager^2^	Unadjusted logistic regression NS		• Support the change	High moderate -Self report -Dependent variable validity not reported -Missing data management not reported
4. Bostrom 2013 [45] BMC Health Services Research	Cross-Sectional Survey	*N* = 1256 Nurses	Hospital, primary care, care of older people, psychiatric care Sweden	Single item - Participate in implementing research-based knowledge in practice^3^ Responses were dichotomized as high or low (high extent = scores of 3 and 4; low extent = scores of 1 and 2).	Point-of-care	Score of 6 items from the QPS Nordic scale reflecting: Social support; Empowering leadership; Fair leadership^3^		Logistic Regression High- versus low-quality leadership on high versus low extent of implementation *p* < 0.005 + OR = 2.0 (CI = 1.4; 2.8)	• Support the change • Encourage • Distribute work fairly	High moderate -Self report -Sample size not justified
5. Cummings 2018 [50]	Cross-sectional survey (data collected at the end of a cross-over study)	*N* = 333 Nurses Managers Healthcare Aids	Nursing homes Canada	Single item for instrumental research use (scoring not stated)	Point-of-care	Score of 11-items from manager support Scale Score of 5-items from coaching conversation scale		Structural equation model (SEM): model chi square (X^2^) Manager support: NS Coaching conversations: NS	• Support the change • Communicate with staff	High moderate -Probability sampling not used -Sample size not justified -Self report
6. Estabrooks 2009 [46] BMC Health Services Research	Cross-sectional survey	*N* = 764 Nurses	Hospital Canada	Single item for instrumental research use scored on 5-point frequency scales from ‘10% or less of the time’ to ‘almost 100% of the time’.	Point-of-care	Score of 6 items from the Alberta Context Tool reflecting emotionally intelligent leadership^4^	Pearson’s correlation (*r* = 0.098) *p* < .05 + (0.098) ANOVA to assess changes in mean value of leadership score with increasing RU scores (test statistic not reported) NS		• Emotionally intelligent leadership behaviors	Strong -Self report -Response rate < 50%
7. Forberg 2014 [47] Worldviews on Evidence-Based Nursing	Cross-sectional survey	*N* = 639 Nurses	Hospital Sweden	Adherence to 3 research-based practices. Scoring was dichotomized for each practice as ‘always’ versus ‘not always’ (represents response alternatives never, rarely, occasionally, frequently)	Point-of-care	Score of 6 items from the Alberta Context Tool reflecting Emotionally intelligent leadership^4^	Logistical regression 1. Disinfection of hands NS 2. Disposable gloves NS 3. Daily inspection NS	Logistic regression 1. Disinfection of hands: NS	• Emotionally intelligent leadership behaviors	Low moderate -Management of missing data not reported -Self report -Sample size not justified -Probability sampling not used
8. Squires 2013 [48] BMC Health Services Research	Cross-sectional survey	*N* = 735 Nurses	Hospital Canada	Single item for instrumental research use scored on 5-point frequency Scales from ‘10% or less of the time’ to ‘almost 100% of the time’.	Point-of-care	Score of 6-items from the Alberta Context Tool reflecting emotionally intelligent leadership^4^		Generalized estimating equations (GEE) Estimate: NS	• Emotionally intelligent leadership behaviors	Strong -Self report -Response rate < 50%
9. Yamada 2017 [49] BMC Health Service Research	Cross-sectional survey	779 Nurses	Hospital Canada	Single item for instrumental research use scored on 5-point frequency scale from 1 = never use to 5 = almost always use	Point-of-care	Score of 6 items from the Alberta Context Tool reflecting emotionally intelligent leadership^4^	Binomial distribution and logit link (analogous to logistical regression) Pain assessment: NS Pain management: NS	Generalized estimating equations (GEE) Estimate: chi square (x^2^) Pain intensity: x^2^ = 7.03; *p* = 0.008	• Emotionally intelligent leadership behaviors	Low moderate -Probability sampling not used -Sample size not justified -Self report -Response rate < 50% -Management of missing data not reported
Mixed methods (*n* = 3)										
Balbale 2015 [51] Implementation Science	Mixed methods Cross-sectional survey and qualitative	*N* = 295 survey *N* = 30 interviews Nurses, allied therapists, physicians, physician assistants	Nursing home USA	Summary score of level of guideline-based practice—facilities categorized as fully implemented or not fully implemented	Senior	3 items from survey developed for the study: 1) Provided adequate staff resources to implement 2) Provided adequate training to implement 3) Provided adequate funding resources to implement	Chi square (x^2^ value not reported) *p* = .0004 84–62% (fully - not fully implemented) *p* = .0042 82–64% (fully - not fully implemented) *p* = .0008 70–43% (fully - not fully implemented)		• Provide resources • Support learning activities	Weak (quant) -Probability sampling not used -Sample size not justified -Response rate < 50% -Self report -Dependent variable reliably and validly not reported -Management of missing data not reported Strong (qual) -Relationship between researcher and participants not reported -Research design not justified
Gifford 2012 [31] Worldviews on Evidence-Based Nursing	Mixed methods Experimental pilot and qualitative	*N* = 88 *N* = 26 interviews Nurses facilitators managers	Community Canada	Documentation of 5 guideline-based practices for diabetic foot ulcers	Point-of-care	3-month leadership intervention consisting of priority setting and planning Interviews	Chi square (x^2^ value not reported) *p* = 0.015 Higher mean number of research based practices in experimental group than control (1.74 vs 2.44)		• Build coalitions • Support the change • Communicate with staff • Encourage • Monitor indicators • Provide resources • Support learning activities • Plan	High moderate (quant) -Sample size not justified -Post-test only -Dependent variable reliably and validly not reported -Management of missing data not reported Strong (qual) -Relationship between researcher and participants not reported
Rangachari 2015 [52] Health Care Management Review	Mixed method Quasi-experimental Chart audit and qualitative text	*N* = 107 Nurses Managers Physicians	Hospital USA	Catheter certification rate = total number of central catheter insertions observed and certified as adhering to components of the guideline-based central line bundle (CLB)	Point-of-care	52-week communication by managers to promote implementation	Statistical tests or *p* value not provided. Changes over time evaluated using difference-in-proportions tests. Catheter certification rate increased 66 to 100% in one unit; 76–100% in the other unit		• Communicate with staff	High moderate (quant) -Probability sampling not used -Sample size not justified -Dependent variable reliably and validly not reported -No comparison strategy Strong (qual) -Relationship between researcher and participants not reported
Qualitative studies (*n* = 19 studies/22 articles)										
1. Angus 2003 [53] Nursing Inquiry	Qualitative	*N* = 61 Nurses Managers	Hospitals Canada	Research-based practice	Point-of-care	Interviews			• Build coalitions • Support the change • Support learning activities	Strong -Relationship between researcher and participants not reported -Ethical issues not mentioned
2. Cheng 2017 [63] Journal of Clinical Nursing	Qualitative	*N* = 56 Nurses Managers Senior leaders Physician	Hospitals China	Evidence-based practice	Point-of-care and senior	Interviews			• Align with organisational mission/vision • Build coalitions • Participate in planning • Support the change • Encourage • Enforce/embed practice in policy • Monitor indicators	Strong -Relationship between researcher and participants not reported
Cheng 2018 [64] journal of nursing management	Qualitative Secondary analysis	*N* = 15 Nurses Managers	Hospitals China	Evidence-based practice	Point-of-care and senior	Interviews				Strong -Relationship between researcher and participants not reported
3. Chimeddamba 2015 [65] Implementation Science	Qualitative	*N* = 40 Nurses Managers Physician	Family health centres Mongolia	Guideline use	Point-of-care	Interviews			• Align with organisational mission/vision • Build coalitions • Participate in planning • Monitor indicators	Strong -Relationship between researcher and participants not reported -Research design not justified
4. Fleiszer 2016 [54] International Journal of Nursing Studies	Qualitative	*N* = 39 Nurses Facilitators Managers	Hospitals Canada	Guideline use	Point-of-care	Interviews			• Align with organisational mission/vision • Participate in planning • Support the change • Communicate with staff • Monitor indicators • Support learning activities	Strong -Relationship between researcher and participants not reported
Fleiszer_2 2016 [66] Journal of Nursing Management	Qualitative	*N* = 39 Nurses Facilitators Managers	Hospitals Canada	Guideline use	Point-of-care	Interviews Observations Document reviews				Strong -Relationship between researcher and participants not reported
5. Gifford (2006) [55] Nursing Leadership	Qualitative	*N* = 17 Nurses Facilitators Managers Senior leaders	Hospitals Nursing home Canada	Guideline use	Point-of-care and senior	Interviews			• Align with organisational mission/vision • Build coalitions • Support the change • Communicate with staff • Encourage • Monitor indicators • Support learning activities	Strong -Relationship between researcher and participants not reported
6. Graham 2004 [56] Birth	Qualitative	*N* = 59 Nurses Facilitators Managers Senior leaders	Hospitals Canada	Guideline use	Point-of-care and senior	Interviews			• Support the change • Embed practice in policy	**Strong** -Relationship between researcher and participants not reported
7. Herbert 2017 [67] BMC Health Services Research	Qualitative	*N* = 26 Nurses Facilitators Managers Allied Health	Hospital England	Evidence-based practice	Senior	Interviews			• Support the change	Strong -Relationship between researcher and participants not reported
8. Higuchi 2017 [68] Journal of Wiley Clinical Nursing	Qualitative	*N* = 132 Nurses Facilitators Managers Senior leaders Healthcare aid Physician Other providers	Hospitals, long-term care, community health agencies, community health centre Canada	Guideline use	Senior	Interviews focus groups			• Support the change • Build coalitions	Strong -Relationship between researcher and participants not reported
9. Ireland 2013 [57] Worldviews on Evidence-Based Nursing	Qualitative	*N* = *95* Nurses Allied Health	Hospitals Canada	Guideline use	Point-of-care	Interviews focus groups			• Support the change • Participate in planning • Monitor indicators	Strong
					Senior				• Support the change • Build coalitions	
10. Kueny 2015 [69] Journal of Healthcare Leadership	Qualitative	*N* = 9 Nurses Managers	Hospitals USA	Evidence-based practice	Point-of-care	Interviews			• Align with organisational mission/vision • Build coalitions • Participate in planning • Communicate with staff • Encourage • Support learning • Activities	Strong -Relationship between researcher and participants not reported
					Senior				Communicate with staff	
11. Matthew-Maich 2012 [59] Journal of Clinical Nursing	Qualitative	*N* = 112 Clients Nurses Facilitators Managers Senior leaders Physicians Midwives	Hospital Canada	Guideline use	Point-of-care	Interviews			• Build coalitions • Support the change • Encourage • Embed in policy • Support learning activities	Strong -Relationship between researcher and participants not reported
Matthew-Maich 2013 [58] Worldviews on Evidence-Based Nursing	Qualitative	*N* = 112 Clients Nurses Facilitators Managers Senior leaders Physicians Midwives	Hospital Canada	Guideline use	Point-of-care	Interviews				Strong -Relationship between researcher and participants not reported
					Senior				• Support the change	
12. Munce 2017 [70] Health Services Research	Qualitative	•N = 33 Nurses, Managers Allied Health	Rehabilitation centres Canada	Guideline use	Point-of-care	Telephone focus groups			• Build coalitions • Support the change	Strong -Relationship between researcher and participants not reported
13. Ploeg 2007 [60] Worldviews on Evidence-Based Nursing	Qualitative	*N* = 125 Nurses, Facilitators Managers Senior leaders Allied Health	Hospitals Community Nursing Home Canada	Guideline use	Point-of-care and Senior	Interviews			• Align with organisational mission/vision • Participate in planning • Support the change • Communicate with staff • Encourage • Embed practice in policy • Provide resources • Support learning activities	Strong -Relationship between researcher and participants not reported
14. Raijmakers 2015 [71] BMJ Supportive & Palliative Care	Qualitative	*N* = 28 Nurses Managers Physicians	Nursing homes and home care organisations Netherlands	Evidence-based practice	Point-of-care	Interviews and focus group			• Support the change	Strong -Relationship between researcher and participants not reported
15. Spyridonidis 2016 [72] British Journal of Management,	Qualitative	*N* = 46 Nurses Managers Physician	Hospitals UK	Guideline use	Point-of-care	Interviews			• Build coalitions	Strong -Relationship between researcher and participants not reported -Unclear if ethical approval was sought
16. Stetler 2014 [5] Worldviews on Evidence-Based Nursing	Qualitative	N = 95 Nurses Managers	Hospitals USA	Evidence-based practice	Point-of-care	Interviews and focus groups			• Support the change • Communicate with staff • Encourage	Strong -Relationship between researcher and participants not reported -Number of participants not clear
					Senior				• Align with organisational mission/vision • Participate in planning • Support learning activities	
17. Sving 2017 [73] Journal of Clinical Nursing	Qualitative	*N* = 36 Nurses Managers	Hospital Sweden	Evidence-based practice	Point-of-care	Interviews and focus groups			• Support the change • Communicate with staff • Provide resources	Strong -Relationship between researcher and participants not reported
18. Van der Zijpp 2016 [61] Worldviews on Evidence-Based Nursing	Qualitative	*N* = 127 Nurses Facilitators Managers	Long-term care England, Netherlands, Republic of Ireland, Sweden	Guideline use	Senior	Interviews			• Align with organisational mission/vision • Support the change	Strong -Relationship between researcher and participants not reported -Recruitment strategy not clear
19. Wallin 2005 [62] International Journal of Nursing Studies	Qualitative	*N* = 45 Nurses	Hospitals Sweden	Guideline use	Point-of-care	Interviews			• Communicate	Strong -Relationship between researcher and participants not reported

*Nurses = e.g. registered nurses, licenced practical nurses, point-of-care nurses, staff nurses, nurse practitioner, lactation consultant; facilitators = e.g. educators, advanced practice nurses, professional practice leaders, change team facilitators, project leads; managers = e.g. nurse managers, supervisors; senior leaders = e.g. administrators, directors, chief executive, allied health; professionals = e.g. physiotherapy, occupational therapy, speech-language pathology, nutritionist, dietitians, rehabilitation professionals

1Responses reported as always, sometimes and never

^2^Scale: 1-strongly disagree, 2-disagree, 3-do not know, 4-agree and 5-strongly agree

^3^Responses ranged from 1-very often or always to 5-seldom or never

_4_Scale: 1-strongly disagree, 2-disagree, 3-neither agree or disagree, 4-agree and 5-strongly agree

The total number of participants in the combined studies was 5840 nursing staff (including nursing assistants or healthcare aids), 332 point-of-care managers, 190 physicians and or other healthcare providers, 129 senior leaders and 110 allied health professionals. Participants of the 11 studies that reported gender [43–50, 57, 63, 64, 69] are as follows: 92% were female and 8% male.

Over half the studies (*n* = 21, 68%) were published in the past 5 years (2013–2018) [5, 45, 47–52, 54, 57–59, 61, 63–73] with the remainder published in the preceding 10 years (2003 and 2012) [31, 42–46, 55, 56, 60, 62]. The earliest study was published in 2003 [39], the number peaked in 2017 (*n* = 6) [49, 63, 67, 68, 70, 73], and two were published in early 2018, when the search ended [50, 64].

### Measures of research use

The dependent variable of instrumental research use was measured through a single-item score on a 5-point frequency scale, capturing how often participants use research-based practices when caring for patients (1 = less than 10%; 5 = almost 100%) [46, 48, 49], (scoring not stated [50]). A mean score of nine items [44] and a single item [43] from the Research Utilization Questionnaire measuring participants’ agreement to using research findings in daily practice on a 5-point Likert scale (1 = strongly disagree to 5 = strongly agree).

Implementation of specific guideline recommendations was the dependent variable in five studies [31, 42, 47, 51, 52]. Forberg et al. [47] measured adherence to six guideline-based practices on a 5-point Likert scale (1 = never to 5 = always) and dichotomized each practice as always or not-always occurring, whereas Ball [42] measured how often participants perceived they followed guideline-based practice at four levels: always (100%), often (50–99%), sometimes (< 50%) and never. In the mixed-methods studies, survey scores [51], observations [52] and chart audits [31] determined the extent of guideline-based practices. Qualitative studies investigated implementation of specific guideline recommendations [54–62] or research-based practices [53].

### Measures of leadership

#### Point-of-care managers

In ten studies, cross-sectional survey data was used to investigate statistical associations between research use and managers’ leadership behaviors: six with point-of-care managers and two with senior managers. Different conceptual aspects of leadership were measured across eight of these studies. Leadership concepts were measured through the Alberta Context Tool (*n* = 3) [46–49], Research Utilization Questionnaire (*n* = 2) [43, 44], QPS Nordic scale (*n* = 1) [45], Managers’ Support and Coaching Conversation scales [50], and a survey specially developed for the study (*n* = 2) [42, 51]. Details of the measures used, statistical effects and direction and magnitude of the effect (if known) are presented in Table 1.

The Alberta Context Tool (ACT) was used to measure leadership in four of the included studies [46–49]. Leadership is measured as a mean score on a 5-point Likert scale of six items measuring the unit-level actions of formal leaders. The six leadership items reflect emotionally intelligent leadership and include: focussing on successes; looking for feedback; calmly handling stress; listening, acknowledging and responding; actively mentoring and coaching, and resolving conflicts [46–48].

The Research Utilization Questionnaire (RUQ) was used to measure leadership in two studies led by the same author [43, 44]. Leadership was measured using a single item on a 5-point Likert scale assessing leadership support for research utilisation. Another study by the same author used the QPS Nordic scale to measure three dimensions of leadership with six items [45]. Scores were dichotomized as high and low-quality leadership based on the dimensions: 1) social support, which involved a willingness to listen and help staff with task-related problems; 2) empowering leadership, which involved encouraging staff; and 3) fair leadership, which involved work-distribution and fair treatment of others.

Two mixed-methods studies involved leadership interventions directed at unit level managers, using qualitative data to provide insights into managers’ leadership behaviors [31, 52]. The three-month intervention in Gifford et al. [31] included planning, developing an action plan and increasing communication with staff; whereas the intervention in Rangachari et al. [52] saw managers engage in weekly communications about central venous lines clinical audit results and processes for change over 52 weeks.

#### Senior managers

Two studies developed surveys that included measures of senior managers’ leadership in implementation of research-based practices [42, 51]. Ball [42] measured leadership support using three items (support for clinical practice, policies and procedures) from a 79-item tool. Similarly, Balbale et al. [51] used three items related to managers’ provision of adequate resources and training (number of survey items not revealed).

### Quality assessment

Of the 31 studies reviewed, 20 were rated as strong (65%) [5, 46, 48, 53–73], seven were high-moderate (23%) [31, 42–45, 50, 52], two (6%) were low-moderate [47, 49] and one (3%) was weak [51]. All 19 qualitative articles [5, 53–73] and two of seven cross-sectional studies rated strong [46, 48]. From the nine cross-sectional studies, five rated high-moderate [42–45, 50], two low-moderate [47, 49], and one weak [51]. Both intervention studies rated high-moderate [31, 52]. Discrepancies in quality assessment mainly related to sample representativeness, response rates, reliability and validity of the dependent variable, and treatment of missing data.

### Associations between leadership and research use

Quantitative studies that evaluated associations between measures of managerial leadership and research use had mixed results. Four leadership measures were statistically significant for point-of-care managers (*support* [43, 45]; *empowering leadership* [45]; *fair leadership* [45]; *emotionally intelligent leadership* [49], and two measures were not (*support* [44, 50]; *emotionally intelligent leadership* [46–48]). For example, Bostrom et al. [43] showed via multivariate analysis (*p* = 0.044) that support from point-of-care managers using the RUQ was significantly related to nurses’ use of research findings; however, in another study using the same instrument, managers’ support was not significantly related to research use in participants who scored as research users compared to non-research users [44]. Using the QPS Nordic scale, higher leadership scores were significantly correlated to increased research use in multivariate analysis (*p* < 0.005) [45]. For senior managers, three leadership measures were statistically significant (*support* [42]; *provide resources* [51]; *provide training* [51]).

Three of four studies that tested an association between leadership measured with the ACT and research use showed non-significant results when more rigorous tests were performed. Estabrooks et al. [46] demonstrated statistically significant (*p* < .05) correlations between research use and leadership scores with Pearson’s correlation; however, a relationship was not demonstrated with an ANOVA measuring increasing levels of research use and leadership scores. Yamada et al. [49] showed that leadership significantly moderated the effect of research use and pain intensity in in hospitalized children.

Studies including a leadership intervention for unit level managers [31, 52] both demonstrated significant differences in research use scores before and after the intervention, with qualitative data providing insights about leadership behaviours used by managers. In the two quantitative studies measuring senior managers’ leadership [42, 51], statistical significance was demonstrated between leadership and research-based practices.

### Leadership behaviours

Twelve leadership behaviours for point-of-care managers were studied in association with research use by clinical staff, and ten for senior managers. Ten of the 12 behaviours for point-of-care managers demonstrated a positive association with research use that were supported by both a qualitative and quantitative or mixed-methods studies. One behaviour that was statistically significant in a quantitative study (*distributes work fairly*) [45] did not emerge in the qualitative data*.* All senior managers’ behaviours emerged from qualitative data with four of those behaviours (40%) also supported by quantitative or mixed-methods studies.

For allied health professionals, three behaviours were identified for point-of-care managers and four behaviours for senior managers. Table 2 provides a complete list of the leadership behaviours studied in association with research use by nurses and allied health professionals. Together, behaviours encompassed change-oriented, relations-oriented and task-oriented leadership behaviours.

**Table 2:** Leadership behaviours studied in association with research use by clinical staff

Point-of-care managers’ leadership behaviour	Quantitative studies (*n* = 8)	Mixed methods (*n* = 2)	Qualitative studies (*n* = 15)	Total no. (*n* = 26)
Change-oriented leadership behaviours				
• Align with organisational mission/vision	–	–	6	6
• Build coalitions with inter-professional colleagues	–	1	8	9
• Participate in planning implementation strategies	–	1	6	7
• Support the change	4 [2+/2−]	1	13	18
Relation-oriented leadership behaviours				
• Communicate with staff	1 [−]	2	8	11
• Encourage	1 [+]	1	7	9
• Emotionally intelligent leadership	4 [1+/3−]	–	–	4
Task-oriented leadership behaviours				
• Embed practices in policy	–	–	3	3
• Distribute work fairly	1 [+]	–	–	1
• Monitor indicators	–	1	5	6
• Provide resources	–	1	3	4
• Support learning activities	–	1	6	7
Senior managers’ leadership behaviours	Quantitative studies (*n* = 1)	Mixed methods (*n* = 1)	Qualitative studies (*n* = 11)	Total no. (*n* = 13)
Change-oriented leadership behaviours				
• Align with organisational mission/vision	–	–	5	5
• Build coalitions with inter-professional colleagues	–	–	4	4
• Participate in planning implementation	–	–	2	2
• Support the change	1 [+]	–	7	8
Relation-oriented leadership behaviours				
• Communicate with staff	–	–	3	3
• Encourage	–	–	2	2
Task-oriented leadership behaviours				
• Embed practice in policies	1 [+]	–	3	4
• Monitor indicators	–	–	1	1
• Provide resources	–	1[+]	1	2
• Support learning activities	–	1 [+]	3	4

*[+]* association statistically significant, *[−]* association not statistically significant

### Change-oriented leadership behaviours

The most commonly cited behaviour for point-of-care and senior managers was *supporting the change* that involved being conceptually and operationally committed to research-based practices [5, 31, 43, 53, 55–61, 64, 70, 71, 73]. Point-of-care managers also ensured that messages about research-based care were consistent with organisational directions and senior leaders’ expectations for performance [54, 55, 60, 63, 65, 66, 69], while senior managers engaged in strategic behaviours to reinforce research-based practices as part of the organisation’s mission or philosophy [5, 55, 60, 63, 72].

Both point-of-care and senior managers built coalitions with inter-professional colleagues, for example, by negotiating with medical staff to change routine orders [53] and working cooperatively with other departments or nurse specialists [31, 55, 57–59, 63–65, 68–70, 72]. Point-of-care managers were involved in planning implementation activities and establishing strategies that aligned to clinical realities so staff could use research evidence in practice [5, 31, 54, 57, 63–66, 69].

### Relations-oriented leadership Behaviours

Point-of-care managers communicated with staff, giving and seeking information about reasons for change, goals to achieve and audit results [52, 54, 55, 61, 62]. They used targeted language about using research evidence in practice [5], encouraged staff to ask questions and voice concerns [45] while incorporating discussions about research-based practices into group shift reports [54, 58]. They provided clear and explicit reasons research-based practice changes would improve practice, addressing individual concerns and actively encouraging staff while acknowledging efforts to change [5, 31, 55, 58, 61, 63, 64, 69].

Relations-oriented leadership behaviours of senior managers emerged in two qualitative studies [55, 60]. Senior managers communicated and encouraged staff by articulating support and addressing concerns about research use in practice**.**

### Task-oriented leadership behaviours

Task-oriented leadership behaviours involved point-of-care and senior managers embedding specific research-based practices into policies [31, 42, 56, 59, 60, 63, 64], providing necessary equipment and supplies [31, 51, 60, 61, 73], supporting learning activities [51, 53–55, 59, 60] and monitoring indicators of research-based practices [31, 54, 55, 57, 63–65]. Distributing work fairly, measured on the QPS Nordic Scale, involved distributing work impartially and treating others equally and was higher in units with more research-based care (*p* < 0.005) [45].

## Discussion

### Summary of findings

This systematic review examined qualitative and quantitative evidence on associations between managers’ leadership behaviours and nurses and allied health professionals’ use of research evidence in clinical practice. Most of the studies were conducted in North America and Europe. No studies focused exclusively on allied health professionals, and only six of the 31 studies reviewed included allied health professionals, offering little empirical evidence for their leadership behaviours that support research use. However, all included studies involved nurses and this body of evidence provides empirical support for a range of leadership behaviors. Twelve leadership behaviors had been studied in association with research use, and 11 of these indicated a positive trend towards influencing professional staff to use research evidence in clinical practice.

Since the 2007 review on managerial leadership for nurses’ use of research evidence [32]), 19 more studies have been published with a greater number of leadership behaviours identified and a stronger association established with research use. It should be remembered that, although the study purposes were similar, this review had different inclusion criteria. In the current review, a statistical link was required between a leadership variable and research use whereas in the 2007 review, descriptions of variables met inclusion. In addition, implementation of research evidence must have explicitly occurred in the qualitative studies in the current review rather than speculatively explored as in the 2007 review. The current review provides more robust evidence for a greater number of leadership behaviours, increasing understanding of the relationship between leadership and research use. For example, in the past 10 years, evidence has emerged on the importance of managers aligning research use with an organisation’s mission, building coalitions with inter-professional colleagues, and being involved in planning implementation strategies. Further evidence has also accumulated on the importance of managers providing support, embedding research evidence in policy and monitoring implementation.

In this synthesis, studies with qualitative (*n* = 19) and mixed-methods (*n* = 3) designs contributed more information about how leaders influenced research use than quantitative studies (*n* = 9). However, data extracted from quantitative studies did not always align with themes extracted from the qualitative data and vice-versa. For example, the measures of *emotionally intelligent leadership* [41, 53, 54] and *fair leadership* [45] emerged in quantitative studies only. Different conceptualizations of leadership in research instruments may partially account for the low number of quantitative studies that provided information on leadership behaviours. The QPS Nordic scale, used by Boström et al. [45], measured three aspects of leadership (social support, encouragement, fair leadership) whereas the Alberta Context Tool (ACT), used by Estabrooks et al. [46], Förberg et al. [47] and Squires et al. [48], had a single score representing emotionally intelligent leadership. While two of the individual items in the ACT leadership subscale aligned with our findings (*communicates with staff and encourages staff*), these items were not individually measured and could not be synthesized separately into our findings. Consistent measurement tools that specifically capture leadership behaviours for research use are necessary to enable meta-analysis in future systematic reviews.

### Multidimensional nature of leadership

Data support the multidimensional nature of leadership and its alignment with behavioural leadership theory [13–15] and concepts of transformational and transactional leadership theory [16, 17]. Transformational leadership is the degree to which a leader inspires and motivates others to follow an ideal or a particular course of action [16], while transactional leadership focuses on incentives and rewards to meet quality standards [17]. Our findings show that managerial leadership, for both point-of-care and senior managers, inspire, encourage and provide incentives for staff through a combination of change, relations and task-oriented behaviours that are responsive to specific clinical contexts and situations. These behaviours are consistent with transformational and transactional leadership approaches and support the multidimensional nature of implementation leadership previously reported [74–76].

### Collaborative activities

The change and relation-oriented behaviours of *building coalitions*, *participating in planning* and *communicating with staff* reveal an interdependent staff/manager relationship. Managers used integrated strategies within and outside their units to build a sense of community and a culture that supports research use. Findings revealed that point-of-care managers do more than encourage staff to conduct specific tasks and follow policies; they also engaged in tailored exchanges within and across departments and disciplines that influenced the work environment and promoted research use. Managers’ priorities and what they pay attention to can signal organisational priorities to staff and directly influence the work environment culture [77].

Our findings highlight managers’ use of collaborative approaches such as building coalitions with inter-professional colleagues, to foster staff’s use of research in routine practice. This involved negotiating, working cooperatively and engaging actively in collaborative activities. A social network analysis in a Canadian public health department found that managers were central to knowledge flow, interactions and inter-personal connections with staff seeking information about practice [78]. With multidisciplinary collaborations’ importance for high-quality outcomes in healthcare settings [79], managers play an important role in fostering these collaborations to support staff use research in clinical practice.

### A common message

Our systematic review provides further evidence of manager/staff dyads being influential in translating research evidence into action [69, 80, 81]. Moreover, leadership for research use extends beyond a leader-follower exchange to include change and task-oriented behaviours that influence the work environment through organisational structures and processes such as *aligning with the organisational mission or vision*, *embedding in policy*, and *providing resources*. This builds on conceptualization of leadership as meso and macro-level activities that influence individuals, the work environment milieu and organisational infrastructure to move towards goals [55, 82, 83].

Schein [77] describes a leader’s focus and how they communicate priorities as ‘primary embedding mechanisms’ which are powerful tools to create a work environment for change. Our qualitative findings highlighted that point-of-care and senior managers aligned the concept of research use to a broader organisational mission or vision, signalling to staff the macro-level leadership support for research use in the organisation. Aarons et al. (2016) similarly showed that coordinated and concerted support from leaders at multiple organisational levels, including a common message that links research use to the organisation’s mission, vision, values, and operations, contributed to successful implementation and sustained research use in social services organisations [9].

### Context of settings

The small heterogeneous sample in this review did not allow for comparisons across countries, professional groups or clinical settings. Leadership behaviours that most frequently emerged were *communication*, *encouragement*, *supporting the change* and *supporting learning activities.* The relevance of our findings to other cultural contexts is, however, unclear, particularly where management and leadership conceptualisations may differ with expressions of individuality and social desirability [84]. For example, integrating indigenous ways of knowing is fundamental to using research in healthcare practices in indigenous communities in Canada and involves the participation of community leaders, chiefs and elders [85]. Indigenous people have a long and established history of translating their own knowledge into actions [86] and managers working with indigenous communities must consider nurses and allied health professional’s use of research evidence within the broader context of colonisation, discrimination and historical trauma. It is unclear how leadership behaviours from this review translate to different global or cultural contexts.

### Inter-professional implications

An increase in published reports over the past 5 years suggests that managerial leadership is gaining attention as an area of study. Note, however, that all studies involved nurses and only six included allied health professionals. While ‘allied health professionals’ can include different professional groups, dependent on where and who is defining them [29], we chose to only focus on physiotherapy, occupational therapy, speech-language pathology, and dietitians as they are central to the delivery of health care services alongside nursing and medicine. While a positive association has been established between leadership and social services workers’ research use in community mental health settings and child welfare social services [87] [9], these studies did not meet inclusion criteria in this review. However, Aarons et al. [9] and Aarons and Sawitzky [87] findings are consistent with ours, demonstrating the full range of leadership behaviours that influence the acceptance and use of research evidence in clinical practice.

With few studies including allied health professionals, little can be extrapolated from the data regarding their managers’ leadership. Although allied health professionals are part of an interdisciplinary team with a professional obligation to incorporate the best available research evidence into their practices, their organisation of care is typically more independent than nurses. Our findings may have limited transferability to leadership directions of managers working with allied health professionals.

### Methodological implications for future research

To increase confidence in future study results examining managerial leadership and research use, methodologies with higher internal and external validity are required. To move the science forward and develop interventions that improve the quality of patient care, five future research implications are noted.

First, research is needed to understand the conceptual similarities and differences between leadership behaviors identified in this review, including studies exploring leadership in different cultural contexts to expand implementation leadership theory. Second, building on conceptual development of leadership for research use, there is a need for consistent measures across studies as only two instruments were used in multiple studies in our review (Research Utilization Questionnaire [43, 44] and Alberta Context Tool [46–48]) and the absence of common measures makes it difficult to build a strong body of knowledge. Using consistent measures will allow findings to be pooled for meta-analysis and sub-group analysis to determine the leadership practices required to facilitate staff use research evidence in different professional groups and sectors.

Third, while our findings are important to understanding how managers and staff perceive leadership for research use, robust methodological studies are now required to determine behaviours that predict nurses’ and allied health professionals’ research use and develop theory-based leadership interventions to improve the quality of patient care. Fourth, since only six studies were found that included allied health professionals and no studies focusing exclusively on them, there is a pressing need for research on managerial leadership with allied health professionals. Finally, studies are needed to understand the conditions that support managers to effectively facilitate and support staff, including conditions that help managers integrate and use research evidence in their management decision-making.

### Limitations

Despite employing rigorous methods in conducting this review, it has limitations that must be acknowledged. While reference lists of included studies were examined for other literature, we did not search gray literature databases, so our search was restricted to primary research in peer-reviewed journals and might have missed relevant unpublished research. Moreover, we did not contact primary authors of excluded studies and may have excluded articles with unclear details of managerial roles. Studies published in languages other than those of the research team (English and French) were also excluded, and databases that could not be accessed in English, such as the Chinese databases CNKI (中国知网) or WANFANG (万方数据库), were not searched. Finally, methodological strengths and weaknesses were not considered while determining our conclusions. Instead, all studies were synthesized equally while reporting on the methodological quality to provide a literature summary and show the current evidence baseline clearly.

## Conclusion

This systematic literature review suggests that managers use a range of leadership practices involving change, relations and task-oriented behaviours to facilitate and support nursing and allied health staff use research evidence use in their clinical practice. While empirical research on allied health professionals is limited, all studies included nurses with a consistent trend across studies that highlighted managers’ commitment, engagement, communication and support. Change-oriented behaviours involve gaining commitment to a broader vision and building coalitions to support the vision, relation-oriented behaviours encompass interpersonal relationships to encourage and support staff, and task-oriented behaviours include concrete activities to operationalize the vision like supporting learning, monitoring performance and outcomes and ensuring policies reflect research-based practices. More robust research designs that include consistent and valid leadership measures specifically for research use are required to advance implementation science on leadership.

## Additional file

Additional file 1: Managerial leadership for research use in nursing and allied health care: search strategies. (PDF 32 kb)

## Abbreviations

ACT: Alberta Context Tool
CASP: Critical Appraisal Skills Programme
LPN: Licensed practical nurse
OTs: Occupational therapists
PTs: Physiotherapists
RN: Registered nurse
RPN: Registered practical nurses
RUQ: Research Utilization Questionnaire
SLPs: Speech-language pathologists
