# Supplementary material for: Managerial leadership for research use in nursing and allied health care professions: a systematic review
Source: Implement Sci. 2018 Sep 27;13:127. doi: 10.1186/s13012-018-0817-7 (PMC6161344; doi:10.1186/s13012-018-0817-7)
Supplement: Supplementary file 1 — Managerial leadership for research use in nursing and allied health care: search strategies. (PDF 32 kb) [file 13012_2018_817_MOESM1_ESM.pdf]

Additional file 1

Managerial leadership for research use in nursing and allied health care: Search strategies

Database: Ovid MEDLINE(R) < 1946 to 2018 June week 1>, Ovid MEDLINE(R) In-Process & Other Non-Indexed Citations < 1946 to 2018 June week 1>

1. Leadership/
2. leader$.tw.
3. manager$.tw.
4. (senior adj2 manag$).tw.
5. (middle adj2 manag$).tw.
6. (Executi$ adj2 manag$).tw.
7. (manag$ adj2 team$).tw.
8. (manag$ adj2 group$).tw.
9. Nursing, Supervisory/
10. Nurse Administrators/
11. (nurs$ adj2 manag$).tw.
12. (nurs$ adj2 leader$).tw.
13. 1 or 2 or 3 or 4 or 5 or 6 or 7 or 8 or 9 or 10 or 11 or 12
14. exp Nurses/
15. exp Nursing Staff/
16. Allied Health Personnel/
17. nurs$.tw.
18. physiotherapist$.tw.
19. dietit$.tw.
20. nutritionist$.tw.
21. (occupational adj2 therapist$).tw.
22. (speech adj2 language adj2 pathologist$).tw.
23. (speech adj2 language adj2 therapist$).tw.
24. (health adj1 care adj1 provider$).tw.
25. (healthcare adj1 provider$).tw.
26. nutritionists/
27. 14 or 15 or 16 or 17 or 18 or 19 or 20 or 21 or 22 or 23 or 24 or 25 or 26
28. (knowledge adj2 Transfer$).tw.
29. (knowledge adj2 translat$).tw.
30. (knowledge adj2 manag$).tw.
31. (knowledge adj2 exchang$).tw.
32. (translation$ adj1 medical adj1 research$).tw.
33. Information Dissemination/
34. Knowledge Management/
35. Evidence-Based Medicine/
36. exp Evidence-Based Practice/
37. (Implement$ adj1 ebm).tw.
38. (Implement$ adj1 ebp).tw.
39. (Implement$ adj1 evidence adj1 based).tw.
40. (evidence adj1 based).tw.
41. (research adj1 implement$).tw.
42. (research adj1 utili?ation).tw.

43. (research adj1 translat$).tw.
44. (research adj1 transfer$).tw.
45. (Implement$ adj2 innovation).tw.
46. (diffusion adj1 innovation$).tw.
47. "Diffusion of Innovation"/
48. (guideline$ adj1 implement$).tw.
49. (knowledge adj2 action).tw.
50. (knowledge adj2 practice).tw.
51. 28 or 29 or 30 or 31 or 32 or 33 or 34 or 35 or 36 or 37 or 38 or 39 or 40 or 41 or 42 or 43 or 44 or 45 or 46 or 47 or 48 or 49 or 50
52. 13 and 27 and 51
53. limit 52 to (english or french)
